# Opting out of Medicare: Characteristics and differences between optometrists and ophthalmologists

**DOI:** 10.1371/journal.pone.0310140

**Published:** 2024-09-09

**Authors:** Michael J. Maywood, Harris Ahmed, Ravi Parikh, Tedi Begaj

**Affiliations:** 1 Department of Ophthalmology, Corewell Health William Beaumont University Hospital, Royal Oak, MI, United States of America; 2 Loma Linda Eye Institute, Loma Linda, CA, United States of America; 3 Manhattan Retina and Eye Consultants, New York, NY, United States of America; 4 Department of Ophthalmology, New York University School of Medicine, New York, NY, United States of America; 5 Associated Retinal Consultants, Royal Oak, MI, United States of America; Nepal Eye Hospital, NEPAL

## Abstract

**Objective:**

To determine the rate of Medicare opt-out among optometrists and ophthalmologists and to contrast the differences in the characteristics and geographic distribution of these populations.

**Design:**

A retrospective cross-sectional study.

**Setting:**

Using a publicly available Centers for Medicare & Medicaid Services (CMS) data set, we collated data for ophthalmologists and optometrists who opted out in each year between 2005 and 2023. We calculated the rate of opt-out annually in each year window and cumulatively from 2005 to 2023. Comparative analysis was used to identify clinician characteristics associated with opt-out.

**Main outcomes and measures:**

Both annual and cumulative rate of ophthalmologist and optometrist opt-out from Medicare.

**Results:**

The estimated prevalence of Medicare opt-outs was 0.52% (77/14,807) for ophthalmologists and 0.38% (154/40,526) for optometrists. Ophthalmologists opting out were predominantly male (67.5%), had a longer practice duration (average 31.8 years), and were more often located in urban areas (83.1%), compared to optometrists (53.2% male, average 19.6 years in practice, 59.1% in urban areas, p = 0.04, p<0.001, p<0.001 respectively). Approximately 83% of ophthalmologists were either anterior segment or oculoplastics specialties, while the majority (52.1%) of optometrists were in optometry-only practices; >75% of identified clinicians were in private practice. Geographical distribution across the US showed variable opt-out rates, with the top 3 states including Oklahoma (3.4%), Arizona (2.1%), and Kansas (1.6%) for ophthalmology and Idaho (4.3%), Montana (3.1%), and Wyoming (1.4%) for optometry.

**Conclusions and relevance:**

Few ophthalmologists and optometrists opt-out of Medicare but this trend has significantly increased since 2012. Of those who disenrolled from Medicare, 83% of ophthalmologists were in urbanized areas while 41% of optometrists were in non-urbanized areas. Because reasons for Medicare opt-out cannot be solely determined by administrative data, further investigation is warranted given the potential impact on healthcare accessibility.

## Introduction

Approximately 19% of the United States population is covered by Medicare health insurance [1], and the Medicare population (aged 65 and older) accounted for 37% of all national health expenditure in 2020 [2]. However, as Medicare reimbursements for physician services have declined, some physicians have disenrolled from Medicare (“opting out”) to remain financially viable [3]. Broadly, “opting out” means that neither the physician nor the beneficiary (i.e. patient) submit a bill to Medicare for services rendered, and instead the beneficiary pays the physician directly out-of-pocket [4]. Reasons for opting out are complex and personal but include a response to specialty-specific reimbursement reductions, avoiding excessive paperwork and billing interface with the government, and receiving full payment at time of service and circumventing cash-flow uncertainty [5].

Recent analyses show that approximately 1.8% of dermatologists [6], 0.6% of otolaryngologists [3], 2.6% of orthopedic surgeons, and 7% of psychiatrists [7] have opted out of Medicare. Ophthalmologists represent a significant percentage of Medicare reimbursement for medical services provided and drug-related costs [8]. Opt-out rates for ophthalmology and optometry have not been published. As the current population ages, ophthalmology is projected to have the 2nd largest workforce shortfall of any medical specialty in the US by 2035 [9], suggesting that even a small fraction of ophthalmologists opting out of Medicare may affect access to care. Medicare is important for providing coverage in rural areas as well as for low-income and geriatric populations [10]. A previous study analyzing data from a national survey from the Centers for Disease Control demonstrated that the rate of physicians accepting new Medicare patients dropped from 96% in 2005 to 93% in 2008 [11].

Medicare opt out may affect patients by potentially decreasing access to care. This study aims to specify the prevalence of clinicians who have opted out of Medicare and characterize clinician demographics, geographic location, and practice models. Furthermore, this work may lead to insight that can be used for regulatory changes to increase Medicare clinician participation.

## Methods

### Data collection

This cross-sectional study was exempt from institutional review board approval because it used publicly available data sets: we examined the Opt-Out Affidavits database from the Centers for Medicare & Medicaid Services (CMS) from available years (2005 to 2023) [4]. The database is maintained by CMS and the US government and is publicly available for download. The CMS database contains information for approximately 15,000 ophthalmologists and 40,000 optometrists. Clinicians who opt out must file procedural paperwork to opt-out of Medicare. Therefore, this database includes all clinicians who have submitted their opt-out forms to CMS. We included all entries for practitioners indicated as ophthalmologists or optometrists on the data collection date, December 1, 2023. The full names, locations (city and state), and date of initial opt-out for each clinician were collected. Through various public domains, we then collected each clinicians’ graduation year from medical school or optometry school, gender, and subspecialty. We stratified ophthalmologists based on their specialty in the following categories: anterior (general ophthalmology, refractive, cornea, and glaucoma), posterior (neuro-ophthalmology, retina, and uveitis), oculoplastics, pediatrics, or unknown. Optometrists were classified as optometry only (solo or group optometry practice), mixed (both ophthalmology and optometry), neuro-optometry/functional or developmental vision or vision rehabilitation, or unknown.

We categorized ophthalmologists by length of practice, calculated as years elapsed since medical school graduation minus five years (to account for four-year residency and one-year fellowship) and optometrist years elapsed since optometry school graduation minus one year (residency) [12]. We then grouped the length of practice as follows: ≤5 years, 6–10, 11–19, 20–29, and ≥30 years. Finally, we determined if the clinician was part of a private practice or academic, university-affiliated institution.

Official US Census metrics, accessed on December 1, 2023, were utilized to determine the population of each clinicians’ listed location, and were stratified as urbanized (>50,000) or non-urbanized (<50,000). The Bureau of Labor Statistics database was utilized to estimate the total number of ophthalmologists [13] (excluding pediatric ophthalmologists) and optometrists [14] currently practicing in the US by state. The prevalence rate was determined by the cumulative number of opted-out clinicians divided by the total number of ophthalmologists and optometrist practicing as of December 2023.

### Statistical analysis

Data analysis was performed using Stata® Statistics/Data Analysis (version 15.1; StataCorp, College Station, Texas). Frequencies and percentages were tallied for categorical variables, and means and standard deviations were calculated for continuous variables. Statistical comparison of the various categorical and continuous variable between optometrist and ophthalmologist were evaluated by mixed-effects linear regression. P-values <0.05 were considered statistically significant.

## Results

A total of 77 ophthalmologists and 154 optometrists were identified as Medicare opt-outs, which represent an estimated prevalence of 0.52% and 0.38%, respectively (Table 1). Clinician characteristics are described in the table. As compared to optometrists, ophthalmologists were more often male (67.5% v. 53.2%, respectively, p<0.04), had been in practice longer (31.8 years v. 19.6, respectively, p<0.001), and were more often located in urban settings (83.1% v. 59.1%, respectively, p<0.001).

**Table 1:** Medicare opt-out rates and characteristics between ophthalmologists and optometrists.

Characteristics	Ophthalmology	Optometry	^#^Beta Coefficient/Odds Ratio	P-Value
**Total Number**	77	154		
**Male**	52 (67.5%)	82 (53.2%)	0.13	0.04
**Years in Practice**				
Average (Std Dev)	31.8 (13.6)	19.6 (12.1)	0.015	<0.01
≤5	3 (3.9%)	7 (9.4%)		
6–10	6 (7.8%)	8 (10.8%)		
11–19	3 (3.9%)	28 (37.8%)		
20–29	17 (22.1%)	12 (16.2%)		
≥30	47 (61.0%)	19 (25.7%)		
Unknown	1 (1.3%)	0 (0%)		
**Practice Environment**				
Private Practice	61 (79.2%)	117 (76.0%)	-0.03	0.35
Hospital-	4 (5.1%)	2 (1.3%)		
affiliated/Academics				
Unknown	12 (15.6%)	35 (22.7%)		
**Practice Setting**				
Urbanized (Population >50,000)	64 (83.1%)	91 (59.1%)	-0.19	<0.01
Non-urbanized	13 (16.9%)	63 (40.9%)		
(Population <50,000)				
**Ophthalmology Specialty**			**Optometry Specialty**	
**Anterior**	46 (59.7%)		**Optometry Only**	79 (51.2%)
**Posterior**	3 (3.9%)		**Mixed**	7 (4.5%)
**Oculoplastics**	17 (22.1%)		**Neuro-cognitive**	27 (17.5%)
**Pediatrics**	10 (13.0%)		**Pediatrics**	0 (0%)
**Unknown**	1 (1.3%)		**Unknown**	41 (26.6%)

# Beta coefficient is reported for continuous variables, while odds ratio is provided for dichotomous or categorical values

**Fig 1:**
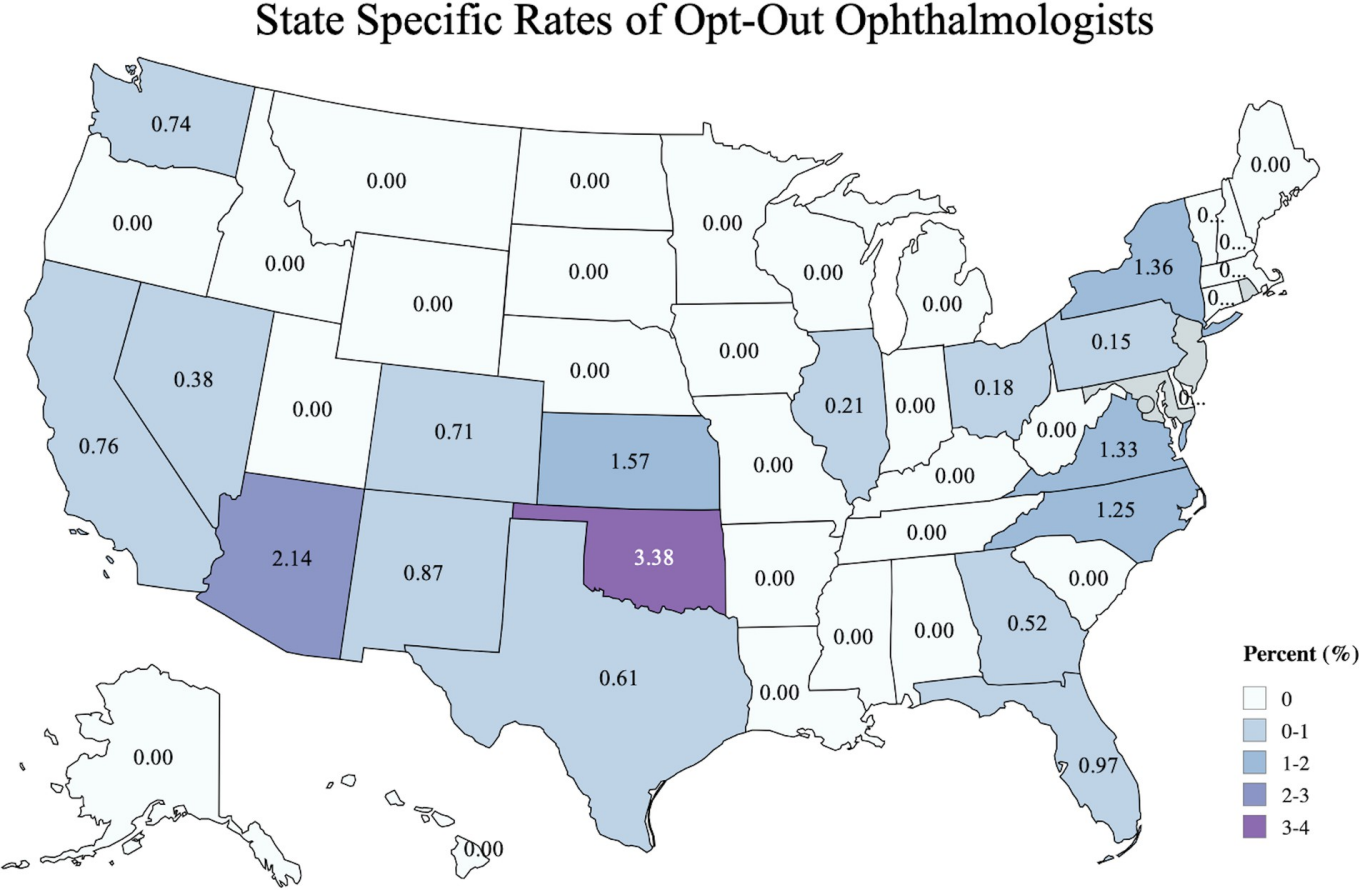
State specific rates of opt-out ophthalmologists. The cumulative opt-out rate for ophthalmologists is shown per US state from 2005 to 2023. Darker colors represent higher turnover rate. (Reprinted from MapChart under a CC BY license, with permission from MapChart, original copyright 2024).

**Fig 2:**
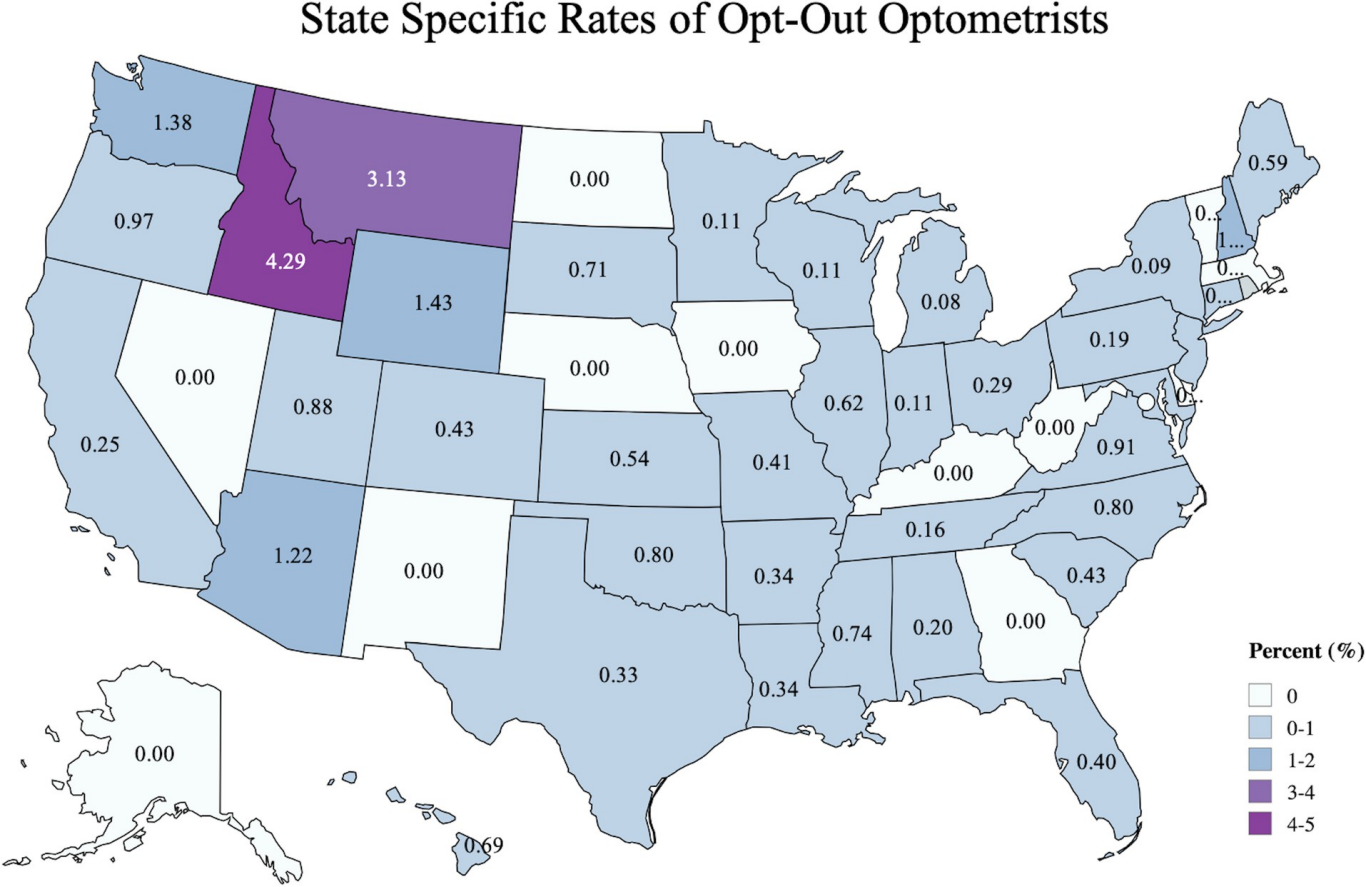
State specific rates of opt-out optometrists. The cumulative opt-out rate for optometrists is shown per US state from 2005 to 2023. Darker colors represent higher turnover rate. (Reprinted from MapChart under a CC BY license, with permission from MapChart, original copyright 2024).

The geographic distribution of US ophthalmologists and optometrists who opted out is depicted in Figs 1 and 2, respectively. Opt-out rates are shown at the state level. For ophthalmologists, the 5 states with the highest opt-out rates were: Oklahoma (3.4%), Arizona (2.1%), Kansas (1.6%), Maryland (1.4%), and New York (1.4%). For optometrists, the top 5 states were: Idaho (4.3%), Montana (3.1%), Wyoming (1.4%), Washington (1.4%), and Arizona (1.3%). States that did not have any clinician opt-out are therefore labeled as zero.

**Fig 3:**
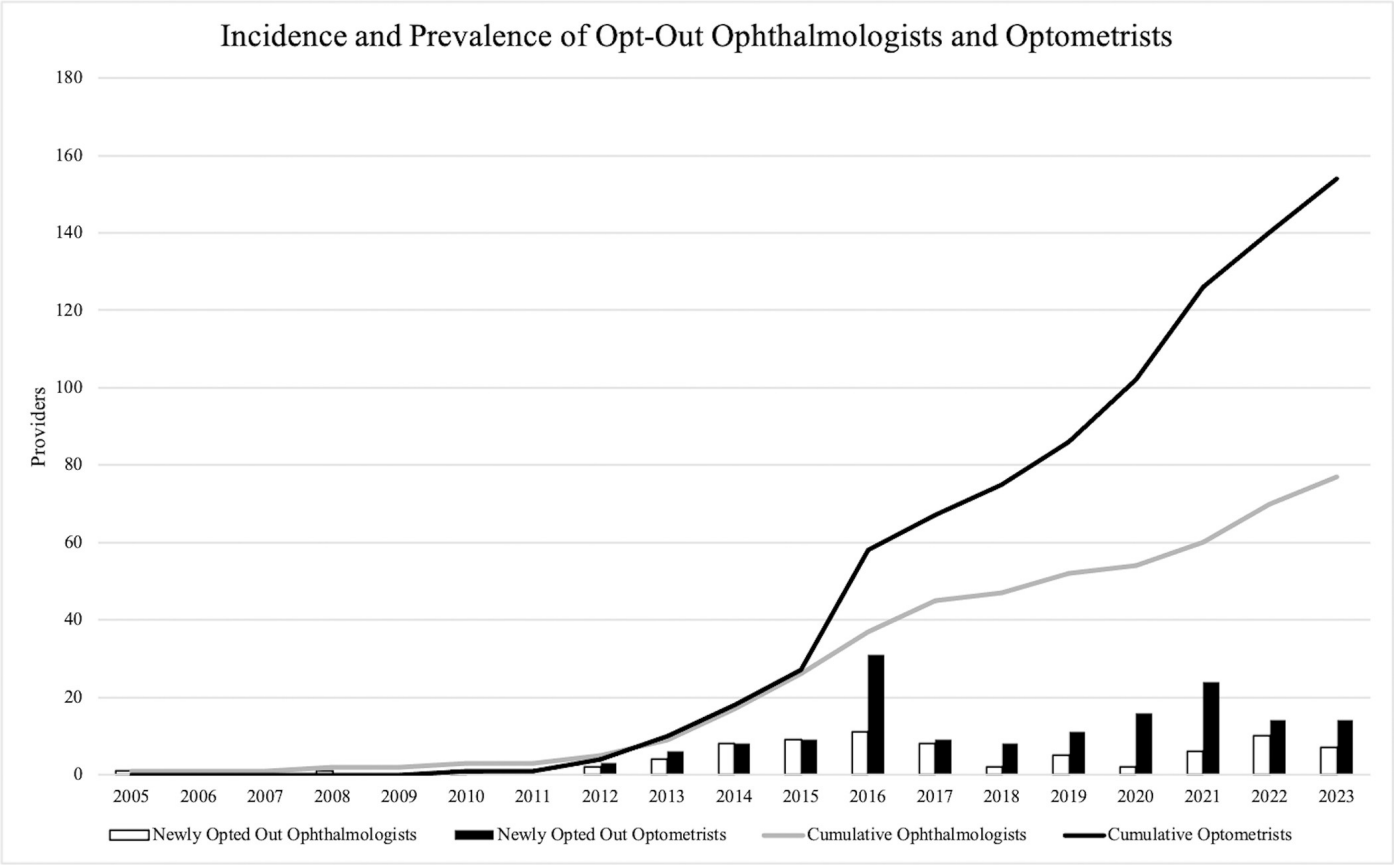
Incidence and prevalence of opt-out ophthalmologists and optometrists. Bar graph shows newly opted out ophthalmologists and optometrists per year, while the line graph demonstrates the cumulative opt-out rate of each clinician type.

Yearly incidence and cumulative prevalence for ophthalmologists and optometrists who have opted out is shown in Fig 3. There were <10 individual ophthalmologists and optometrists who opted out from 2005 to 2012. However, starting in 2012 and onward, there was a significant rise in both ophthalmologist and optometrist opt-out, with a cumulative prevalence of 77 ophthalmologists and 154 optometrists as of December 2023.

## Discussion

In this cross-sectional analysis, 77 (0.52%) ophthalmologists and 154 (0.38%) optometrists have currently opted out of Medicare. Our estimated opt-out prevalence for ophthalmologists is comparable to some specialties like otolaryngology [3] (0.5%), and much lower than others, such as dermatology [6] (1.8%), and psychiatry [7] (7.0%). To our knowledge, this is the first study to determine the prevalence of eye care clinicians who have opted out of Medicare in addition to examining the differences in characteristics between optometrists and ophthalmologists who have opted out.

We observed that >80% of ophthalmologists who opted out performed either anterior segment or oculoplastic surgery. For these physicians, some surgical procedures (e.g. laser vision correction, cosmetic blepharoplasty) are often not covered by insurance and are paid out-of-pocket. Additionally, pediatric ophthalmologists do not usually provide services for Medicare patients, so they may opt out.

The various factors leading clinicians to opt out of Medicare is beyond the scope of this article; each persons’ decision to opt out of Medicare is likely unique and multifactorial. Physicians have historically attempted to increase clinical volume to offset reimbursement cuts to the Medicare Fee Schedule; however, volume cannot be increased indefinitely [5]. Certain clinicians may also benefit from the freedom of being able to set the price of services rendered as they deem appropriate and to deal entirely in cash transactions. Some clinicians may utilize a business model that cuts overhead and personnel costs by limiting paperwork and required office staff. With increased complexity in medical billing, some clinicians may fear increased scrutiny from the government in its attempt to curtail fraud. Finally, clinicians who wish to offer non-traditional or potential “holistic services” may find it beneficial to entirely avoid the recurrent need for advance beneficiary notices in the Medicare patient population [5].

We observed that while the overall proportion of opted-out ophthalmologists and optometrists is similar, there are differing group characteristics. A higher percentage of males with significant clinical experience opted out in ophthalmology as compared to optometry. Nationally, the ratio of older (>55) to younger (<55) ophthalmologists has increased from 0.37 to 0.82 across all practice environments, including metropolitan and rural settings [15]. This trend was also observed in psychiatry, with older psychiatrists opting out more frequently [7]. It is possible that these established physicians may opt out of all insurance plans (including Medicare) and pursue financial reimbursements directly from patients. Whether higher opt-out rates are due to lower reimbursement, increased administrative burden, or other personal or financial reasons, we have observed (in tandem with other medical subspecialties) a modest but increasing rate of physician Medicare opt out rates in the last decade. With a goal of expanding health insurance coverage in the United States, policy makers must be aware of the increasing rates of opt out in order to implement policies to potentially decrease rates in the future.

A secondary finding of our work was the difference in opt-out rates between urbanized and non-urbanized areas. Approximately 83% of ophthalmologists opted out in urban areas, with 45% representing 3 densely populated urban states (California, Texas, and New York). Historical data has shown that physicians who opt out are found in large urbanized communities and are rarely the only available source of care [5]. In contrast, we observed that 41% of optometrists opted out of rural areas; 17% were located in 2 rural states: Montana (3.1% of all optometrists) and Idaho (4.3%). While the overall density of optometrists nationwide has increased from 11.1 to 16.2 per 100,000 people, coverage remains low in rural communities (6.8 optometrists per 100,000) compared to metropolitan communities (16.4 optometrists per 100,000) [15]. These trends may lead to compromised care, particularly in vulnerable settings (e.g. lower socioeconomic status [16]), as smaller rural communities have worse vision outcomes and lower rates of crucial ophthalmic care and procedures [17]. Further work is required to understand what specific factors are causing clinicians to opt-out so that regulatory leaders can potentially modulate these effects in a way that encourages broad Medicare participation. One potential factors that must be examined is Medicare reimbursement so it remains financially feasible for physicians to accept Medicare for services rendered.

The work herein has several limitations. First, the analysis is cross-sectional and cannot determine a causal effect. Second, the opt-out process can be laborious and must be repeated every two years, so we may be underestimating the number of ophthalmologists and optometrists who do not accept Medicare insurance but have not formally opted out. The CMS data provides yearly individual clinician opt-out numbers; however, we were unable to determine the overall yearly rate of clinician opt-out as the number of yearly practicing US ophthalmologists started being published in May 2019 by the US bureau of labor statistics [13]. Third, the actual working years of optometrists and ophthalmologists was not often readily available and years since graduation is only an approximation. Additionally, the number of optometrists for whom data was unknown was higher than ophthalmologists, and it is possible the characteristics and demographics of those missing may affect our observed patterns.
